# Clinical application of Azithromycin in treating pediatric Mycoplasma Pneumonia and its effects on platelet count, D-Dimer Levels, and Serum Inflammatory Factors

**DOI:** 10.12669/pjms.41.12.12489

**Published:** 2025-12

**Authors:** Kun Dong, Wenjuan Tong, Songjian Lu, Honghua Lin

**Affiliations:** 1 Kun Dong, Department of Clinical Immunology, Anhui Children’s Hospital, Hefei 233000, Anhui, China; 2 Wenjuan Tong, Department of Clinical Immunology, Anhui Children’s Hospital, Hefei 233000, Anhui, China; 3 Songjian Lu, Department of Clinical Immunology, Anhui Children’s Hospital, Hefei 233000, Anhui, China; 4 Honghua Lin, Department of Clinical Immunology, Anhui Children’s Hospital, Hefei 233000, Anhui, China

**Keywords:** Azithromycin, Pediatric mycoplasma pneumonia, Platelet count, D-dimer, Inflammatory factors

## Abstract

**Objective:**

To investigate the clinical efficacy of azithromycin in treating pediatric mycoplasma pneumonia and its effects on platelet count, D-dimer levels, and serum inflammatory factors.

**Methodology:**

This was a retrospective study. Eighty children with mycoplasma pneumonia admitted to Anhui Children’s Hospital between June 2022 to June 2024 were selected as study subjects. They were divided into an observation group(n=40) receiving azithromycin and a control group(n=40) receiving erythromycin according to different treatment strategies. Clinical efficacy, time to resolution of symptoms, platelet count, D-dimer levels, serum inflammatory factor levels, and incidence of adverse reactions were compared between the two groups.

**Results:**

The overall response rate of the observation group(95.00%) was significantly higher than that of the control group(72.50%)(χ^2^=7.440,*P*<0.05). The time to fever subsidence, cough resolution, hospitalization duration, and disappearance of pulmonary moist rales in the observation group were all significantly shorter than those in the control group(*t*=14.208,9.639,13.628,31.677, *P*<0.05). After treatment, the platelet count(PLT) and D-dimer(D-D) levels in the observation group were significantly lower than those in the control group (t=3.079, 7.847, P<0.05 for both). The serum levels of TNF-α, CRP, and IL-6 in the observation group were also significantly lower than those in the control group(*t*=6.540,10.406,9.688,*P*<0.05). The incidence of adverse reactions in the observation group (5.00%) was significantly lower than that in the control group(20.00%) (χ^2^=4.114, *P*<0.05).

**Conclusion:**

Azithromycin offers multiple benefits in treating pediatric mycoplasma pneumonia, including rapid symptom resolution, potent anti-inflammatory effects, regulation of coagulation-fibrinolysis balance, reduction in serum inflammatory factor levels, and a lower incidence of adverse reactions.

## INTRODUCTION

Pediatric mycoplasma pneumonia, a common respiratory infectious disease in children caused by Mycoplasma pneumoniae, has seen a global rise in incidence.^1^ As a cell-wall-deficient microorganism, Mycoplasma pneumoniae is inherently insensitive to traditional antimicrobial agents targeting the bacterial cell wall, such as penicillins and cephalosporins, thus limiting clinical treatment options.^2^ In recent years, azithromycin has emerged as a first-line therapy for pediatric mycoplasma pneumonia owing to its unique antibacterial mechanism, favorable pharmacokinetics, and high safety margin.^3^ Blood Platelets (PLT) play a crucial role in the body’s hemostasis and inflammatory response, with changes in their count directly reflecting the body’s pathophysiological status.^4^ D-dimer (D-D), as an important marker of the body’s fibrinolytic activity, exhibits level changes that may be closely associated with alterations in inflammatory responses and coagulation mechanisms during azithromycin treatment.^5^

Additionally, as an infectious disease, mycoplasma pneumonia is characterized by the cascading release of multiple inflammatory cytokines throughout its course.^6^,^7^ However, limited research has addressed the clinical efficacy of azithromycin and its impact on coagulation-inflammatory biomarkers in pediatric patients. Based on this, the present study enrolled 80 children with pediatric mycoplasma pneumonia to investigate the clinical application of azithromycin in treating pediatric mycoplasma pneumonia.

## METHODOLOGY

This was a retrospective study. Eighty children with mycoplasma pneumonia admitted to Anhui Children’s Hospital between June 2022 to June 2024 were selected as study subjects. Subjects were divided into an observation group (40 cases) and a control group (40 cases) based on different treatment strategies.

### Ethical Approval:

The study was approved by the Institutional Ethics Committee of Anhui Children’s Hospital (No.: EYLL-2023-027; date: September 27, 2023), and written informed consent was obtained from the guardians of the participants.

### Inclusion criteria:

- Met the diagnostic criteria for mycoplasma pneumonia.^8^
- Age 3~12 years.
- No recent treatment with related medications within the past month.
- clear consciousness.
- The child’s family members fully understood the study content and signed the informed consent.

### Exclusion criteria:

- Presence of other serious physical or mental disorders.
- Congenital respiratory system disease.
- Allergy to the study drugs.
- Withdrawal from the study due to individual reasons.

There were no significant differences in baseline data (gender, age, disease duration, BMI, and severity) between the two groups (*P*>0.05). Detailed data are presented in Table-I. The control group received erythromycin treatment (manufacturer: Dalian Merro Pharmaceutical Factory, NMPA approval No.: H21021678, specification: 0.25g/vial). The daily dosage was calculated based on the child’s weight at 30 mg/(kg·d), dissolved in 5% glucose solution and administered via intravenous infusion for seven consecutive days. Concomitant use of any other antibiotics was prohibited throughout the treatment period. The observation group received azithromycin treatment (manufacturer: Hainan Poly Pharm. Co., Ltd., NMPA approval No.: H20173261, specification: 0.5g/vial). The daily dosage was similarly calculated based on weight at 10 mg/(kg·d), dissolved in 5% glucose solution and administered via intravenous infusion for seven consecutive days. No other antibiotics were permitted during the treatment course.

**Table-I:** Comparison of Baseline Data (*Χ̅±S*, n%).

Group	Number of cases	Gender		Age (years old)	Disease Duration (d)	BMI (kg/m^2^)	Severity		
		Male	Female				Mild	Moderate	Severe
Observation Group	40	24 (60.00)	16 (40.00)	6.55±2.35	6.58±1.36	17.34±2.59	11 (27.50)	21 (52.50)	8 (20.00)
Control Group	40	27 (67.50)	13 (32.50)	6.43±2.60	6.65±1.14	16.95±2.73	13 (32.50)	18 (45.00)	9 (22.50)
*t/χ^2^ Value*		0.487	0.487	0.225	0.267	0.656	0.238	0.450	0.075
*P Value*		0.485	0.485	0.822	0.790	0.514	0.626	0.502	0.785

### Evaluation Indicators:

### Efficacy evaluation^9^:

After completing the full treatment course, all clinical symptoms completely resolved, and lung X-ray examination showed complete resolution of pre-existing shadows or lesions. Effective: Clinical symptoms improved but did not completely resolve, with residual shadows or lesions still observable on lung X-ray examination. Ineffective: No significant improvement in clinical symptoms was noted, and lung X-ray findings remained unchanged after treatment. Overall response rate = (marked effective + effective)/total cases × 100%.

### Time to resolution of symptoms:

This included fever resolution time, cough resolution time, hospitalization duration, and pulmonary moist rales disappearance time.

### PLT count and D-Dimer levels:

Measured before and after treatment. Measurements were taken before and after treatment. The PLT count was accurately determined using a hematology analyzer; the D-Dimer levels were measured using a high-sensitivity colloidal gold immunochromatographic assay.

### Serum inflammatory factors:

A 3-mL fasting peripheral venous blood sample was collected from each child before and after treatment. The levels of Tumor Necrosis Factor -α (TNF-α), C-Reactive Protein (CRP), and Interleukin-6 (IL-6) were measured using the ELISA method.

### Adverse reactions:

These included nausea, vomiting, dizziness, abdominal pain, diarrhea, and fever. The overall adverse reaction rate was calculated.

### Statistical analysis:

Statistical data were first entered into Excel (2010) spreadsheets and then analyzed using SPSS Statistics (version 22.0). The sample size is estimated by 95% confidence interval. Measurement data (age, disease duration, BMI, etc.) were expressed as () and analyzed using independent samples *t*-tests. Enumeration data (gender, disease severity, etc.) were presented as percentage (%) and evaluated using Chi-square (χ^2^) tests. A *P*-value <0.05 was considered a statistically significant difference.

## RESULTS

**Fig.1:**
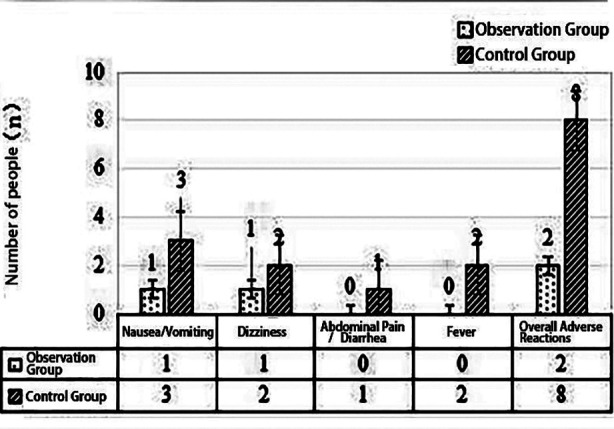
Comparison of Therapeutic Efficacy (n, %).

The overall efficacy of the observation group (95.00%) was significantly higher than that of the control group (72.50%) (P<0.05). Detailed data can be found in Table-II and Fig.1.

**Table-II:** Comparison of Therapeutic Efficacy (n, %).

Group	Number of cases	Markedly Effective	Effective	Ineffective	Overall Effective Rate
Observation Group	40	30 (75.00)	8 (20.00)	2 (5.00)	38 (95.00)
Control Group	40	8 (20.00)	21 (52.50)	11 (27.50)	29 (72.50)
*χ^2^value*					7.440
*P value*					0.006

The observation group demonstrated significantly shorter fever resolution time, cough resolution time, hospitalization duration, and pulmonary moist rales disappearance time compared with the control group (*P*<0.05). Detailed data are presented in Table-III.

**Table-III:** Comparison of Time to Resolution of Symptoms (*Χ̅±S*, d).

Group	Number of cases	Fever Resolution Time	Cough Resolution Time	Hospital Stay Duration	Pulmonary Moist Rales Disappearance Time
Observation Group	40	3.30±0.69	9.03±1.17	9.28±1.36	6.08±0.27
Control Group	40	5.63±0.77	11.53±1.15	13.18±1.20	8.10±0.30
*t value*		14.208	9.639	13.628	31.677
*P value*		0.000	0.000	0.000	0.000

**Fig.2:**
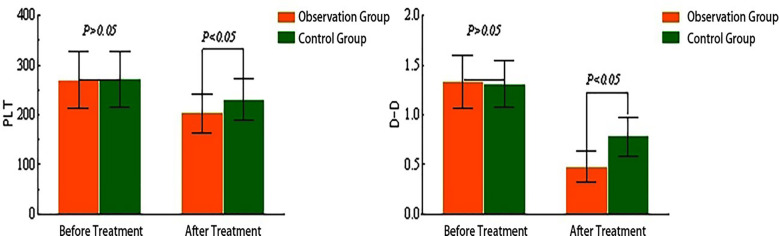
Comparison of PLT Count and D-Dimer Levels ().

Before treatment, there were no significant differences in PLT count or D-dimer levels between the two groups (*P*>0.05). After treatment, both PLT counts and D-Dimer levels decreased in both groups; however, the observation group exhibited significantly lower PLT counts and D-Dimer levels compared to the control group (*P*<0.05). Detailed data are provided in Table-IV and Fig.2.

**Table-IV:** Comparison of PLT Count and D-Dimer Levels ()

Group	Number of cases	PLT (×10^9^/L)		D-D (mg/L)	
		Before Treatment	After Treatment	Before Treatment	After Treatment
Observation Group	40	269.81±56.53	203.32±39.23	1.33±0.27	0.48±0.15
Control Group	40	271.75±55.71	231.31±42.02	1.31±0.23	0.78±0.19
*t value*		0.155	3.079	0.357	7.847
*P value*		0.878	0.003	0.722	0.000

Before treatment, there were no significant differences in serum inflammatory marker levels between the two groups (P>0.05). After treatment, serum inflammatory marker levels decreased in both groups, but the levels of TNF-α, CRP, and IL-6 in the observation group were significantly lower than those in the control group (*P*<0.05). Detailed data are summarized in Table-V.

**Table-V:** Comparison of Serum Inflammatory Markers (*Χ̅±S*)

Group	Number of cases	TNF-α (µg/L)		CRP (mg/L)		IL-6 (pg/L)	
		Before Treatment	After Treatment	Before Treatment	After Treatment	Before Treatment	After Treatment
Observation Group	40	36.07±5.52	5.58±2.11	22.43±1.73	7.08±1.31	61.71±5.36	26.06±5.24
Control Group	40	35.82±5.17	9.57±3.23	22.52±1.71	10.32±1.47	61.62±5.44	37.39±5.22
*t value*		0.209	6.540	0.234	10.406	0.075	9.688
*P value*		0.835	0.000	0.816	0.000	0.941	0.000

**Fig.3:**
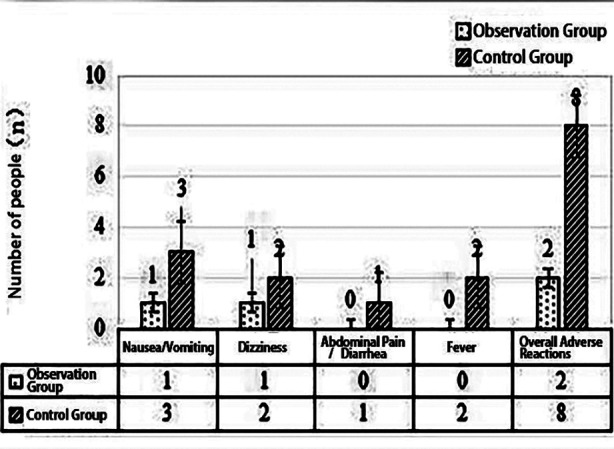
Comparison of Adverse Reactions (n, %).

The overall adverse reaction rate in the observation group (5.00%) was significantly lower than that in the control group (20.00%) (*P*<0.05). Detailed data are presented in Table-VI and Fig.3.

**Table-VI:** Comparison of Adverse Reactions (n, %)

Group	Number of cases	Nausea/Vomiting	Dizziness	Abdominal Pain/Diarrhea	Fever	Overall Adverse Reactions
Observation Group	40	1 (2.50)	1 (2.50)	0 (0.00)	0 (0.00)	2 (5.00)
Control Group	40	3 (7.50)	2 (5.00)	1 (2.50)	2 (5.00)	8 (20.00)
*χ^2^ value*						4.114
*P value*						0.043

## DISCUSSION

The results of this study indicate that the overall efficacy in the observation group (95.00%) was significantly higher than that in the control group (72.50%). The reasoning could be attributed to Azithromycin’s status as a macrolide antibiotic with superior penetration capabilities, allowing it to permeate lung tissues more effectively, thus displaying higher antibacterial activity against Mycoplasma pneumoniae. Azithromycin’s prolonged half-life enables sustained therapeutic drug levels in the body, continuously suppressing pathogen growth and promoting recovery. Comparatively, Erythromycin, although effective, may not have the same tissue penetration and half-life as Azithromycin, resulting in relatively weaker treatment effects. International studies have similarly indicated^10^ that Azithromycin achieves higher cure rates and reduced recurrence rates in treating respiratory infections caused by Mycoplasma. Mycoplasma pneumoniae, an acute respiratory infectious disease with complex pathogenesis, presents a variety of clinical manifestations such as fever, cough, and dyspnea.^11^ In early childhood, the immune system is not fully developed, rendering children less resistant to pathogens. Additionally, the respiratory tissues are more vulnerable, which can lead to more severe and rapidly progressing illness upon infection with Mycoplasma.^12^ Clinical practice has demonstrated^13^ that Azithromycin can effectively inhibit the growth of Mycoplasma pneumoniae, thereby alleviating clinical symptoms like cough, fever, and tachypnea in pediatric patients. Azithromycin’s unique pharmacokinetic properties allow it to maintain effective tissue concentration after a single dose, which is particularly crucial for the continuous antibacterial action required in treating pediatric Mycoplasma pneumonia.^14^

Study findings demonstrate that the time to fever resolution, cough cessation, discharge, and disappearance of lung rales was significantly shorter in the observation group than in the control group. The potential reason could be Azithromycin’s ability to rapidly reach therapeutic concentrations with its rapid onset, promptly inhibiting Mycoplasma growth. This rapid effect significantly reduces ongoing stimulation from pathogens on the organism, expediting the resolution of inflammatory processes. Multiple domestic and international studies^15^ have found that Azithromycin quickly alleviates pediatric symptoms of Mycoplasma pneumoniae such as fever and cough. Its direct action on lung tissues also contributes to the quicker amelioration of symptoms. Azithromycin effectively attenuates pulmonary inflammation and edema, facilitating the restoration of alveolar and bronchial function.^16^ This lung-protective action helps expedite the disappearance of clinical signs like rales, therefore shortening pediatric patient hospitalization duration. Moreover, Azithromycin assists in sputum clearance, further enhancing respiratory status in children.

Post-treatment results show that PLT count and D-D levels in the observation group were significantly lower than in the control group. This might be due to Azithromycin’s multifaceted anti-inflammatory mechanisms reducing pro-inflammatory factor release, thereby decreasing platelet activation and aggregation, which subsequently lowers PLT counts. Reduction in D-D levels reflects Azithromycin’s beneficial impact on coagulation-fibrinolysis system balance. Studies abroad^17^ have noted that Azithromycin modulates coagulation and fibrinolysis system equilibrium, diminishing unnecessary clotting reactions and thrombogenesis. Such precise regulation of the coagulation-fibrinolysis balance aids in maintaining vascular patency and stable blood flow.

The study indicates significant reductions in TNF-α, CRP, and IL-6 levels in the observation group compared to the control group post-treatment. Azithromycin might suppress pro-inflammatory cytokine production directly or indirectly, in addition to its antibacterial action. Multiple studies both domestically and internationally^18^ have corroborated Azithromycin’s ability to markedly reduce levels of TNF-α, CRP, and IL-6, which play crucial roles in inflammatory responses. The reduction in these cytokines signifies attenuation of the inflammatory response and improvement in immune status. One of Azithromycin’s key mechanisms is its immunomodulatory effect, lowering serum inflammatory cytokine levels. It regulates immune response, enhancing anti-inflammatory mediator production or inhibiting pro-inflammatory mediator activity; this modulation helps balance immune reactions, preventing excessive inflammatory damage and promoting self-repair and recuperative capacities.^19^

The study results indicate the overall adverse reaction rate in the observation group (5.00%) was significantly lower than that in the control group (20.00%). Azithromycin’s ability to maintain effective drug concentrations for extended periods reduces the need for frequent dosing, not only improving therapeutic efficacy but also lowering the risk of adverse reactions due to frequent dosing. In comparison to other antibiotics like Erythromycin, Azithromycin exerts less gastrointestinal irritation. Erythromycin often causes nausea, vomiting, and abdominal discomfort during treatment, while Azithromycin is associated with fewer such side effects, thereby enhancing pediatric patient tolerability and compliance. Studies abroad^19^ demonstrate that treatment of pediatric Mycoplasma pneumoniae with Azithromycin shows significant efficacy with a lower incidence of adverse effects compared to traditional antibiotics like Erythromycin. These studies, by comparing adverse reaction rates in different drug groups, confirm the safety advantage of Azithromycin. Other studies have also indicated^20^ that Azithromycin is not only effective but also has mild adverse reactions when treating children’s respiratory infections, making it a suitable first-choice antibiotic.

### Limitations:

It includes a relatively small sample size, which may affect the generalizability of results, and the lack of in-depth exploration of specific mechanisms. Future research should expand the sample size and utilize more advanced methodologies to deeply probe its mechanisms to further verify and optimize this therapeutic regimen.

## CONCLUSIONS

Azithromycin offers multiple benefits in treating pediatric mycoplasma pneumonia, including rapid symptom resolution, potent anti-inflammatory effects, regulation of coagulation-fibrinolysis balance, reduction in serum inflammatory factor levels, and a lower incidence of adverse reactions.

### Authors’ Contributions:

**DK:** Conceived, designed and did statistical analysis and editing of manuscript..

**TW:** Literature search, did data collection and manuscript writing.

**LS** and **LH:** Did review and final approval of manuscript.

All authors have read, approved the final manuscript and are responsible for the integrity of the study.
